# Characterisation of the redox centers of ethylbenzene dehydrogenase

**DOI:** 10.1007/s00775-021-01917-0

**Published:** 2021-11-29

**Authors:** Corina Hagel, Bärbel Blaum, Thorsten Friedrich, Johann Heider

**Affiliations:** 1 Labor für Mikrobielle Biochemie and Synmikro Zentrum für Synthetische Mikrobiologie, Philipps Universität Marburg, 35043 Marburg, Germany; 2 Institut für Biochemie, Albert-Ludwigs Universität, Albertstr. 21, 79104 Freiburg im Breisgau, Germany

**Keywords:** Ethylbenzene dehydrogenase, Electron paramagnetic resonance, Molybdenum cofactor, Iron–sulfur cluster, Heme *b*, Redox potential

## Abstract

Ethylbenzene dehydrogenase (EbDH), the initial enzyme of anaerobic ethylbenzene degradation from the beta-proteobacterium *Aromatoleum* *aromaticum*, is a soluble periplasmic molybdenum enzyme consisting of three subunits. It contains a Mo-*bis*-molybdopterin guanine dinucleotide (Mo-*bis*-MGD) cofactor and an 4Fe–4S cluster (FS0) in the α-subunit, three 4Fe–4S clusters (FS1 to FS3) and a 3Fe–4S cluster (FS4) in the β-subunit and a heme *b* cofactor in the γ-subunit. Ethylbenzene is hydroxylated by a water molecule in an oxygen-independent manner at the Mo-*bis*-MGD cofactor, which is reduced from the Mo^VI^ to the Mo^IV^ state in two subsequent one-electron steps. The electrons are then transferred via the Fe–S clusters to the heme *b* cofactor. In this report, we determine the midpoint redox potentials of the Mo-*bis*-MGD cofactor and FS1–FS4 by EPR spectroscopy, and that of the heme *b* cofactor by electrochemically induced redox difference spectroscopy. We obtained relatively high values of > 250 mV both for the Mo^VI^–Mo^V^ redox couple and the heme *b* cofactor, whereas FS2 is only reduced at a very low redox potential, causing magnetic coupling with the neighboring FS1 and FS3. We compare the results with the data on related enzymes and interpret their significance for the function of EbDH.

**Graphical abstract:**

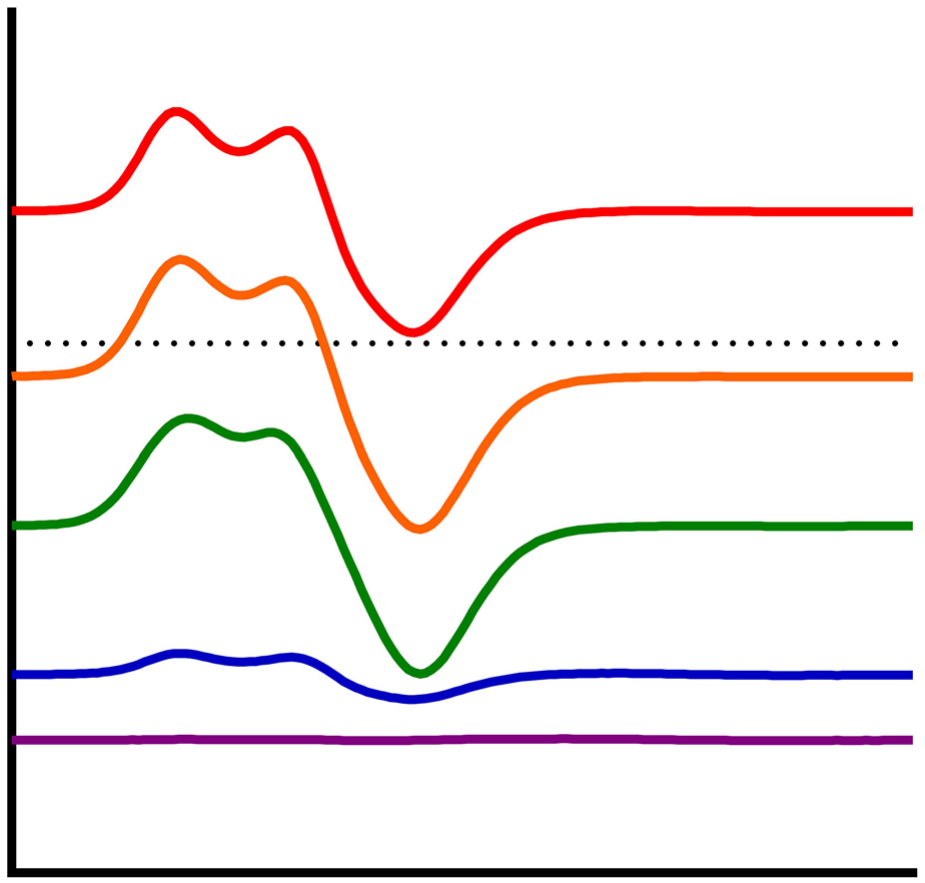

## Introduction 

**Fig. 1:**
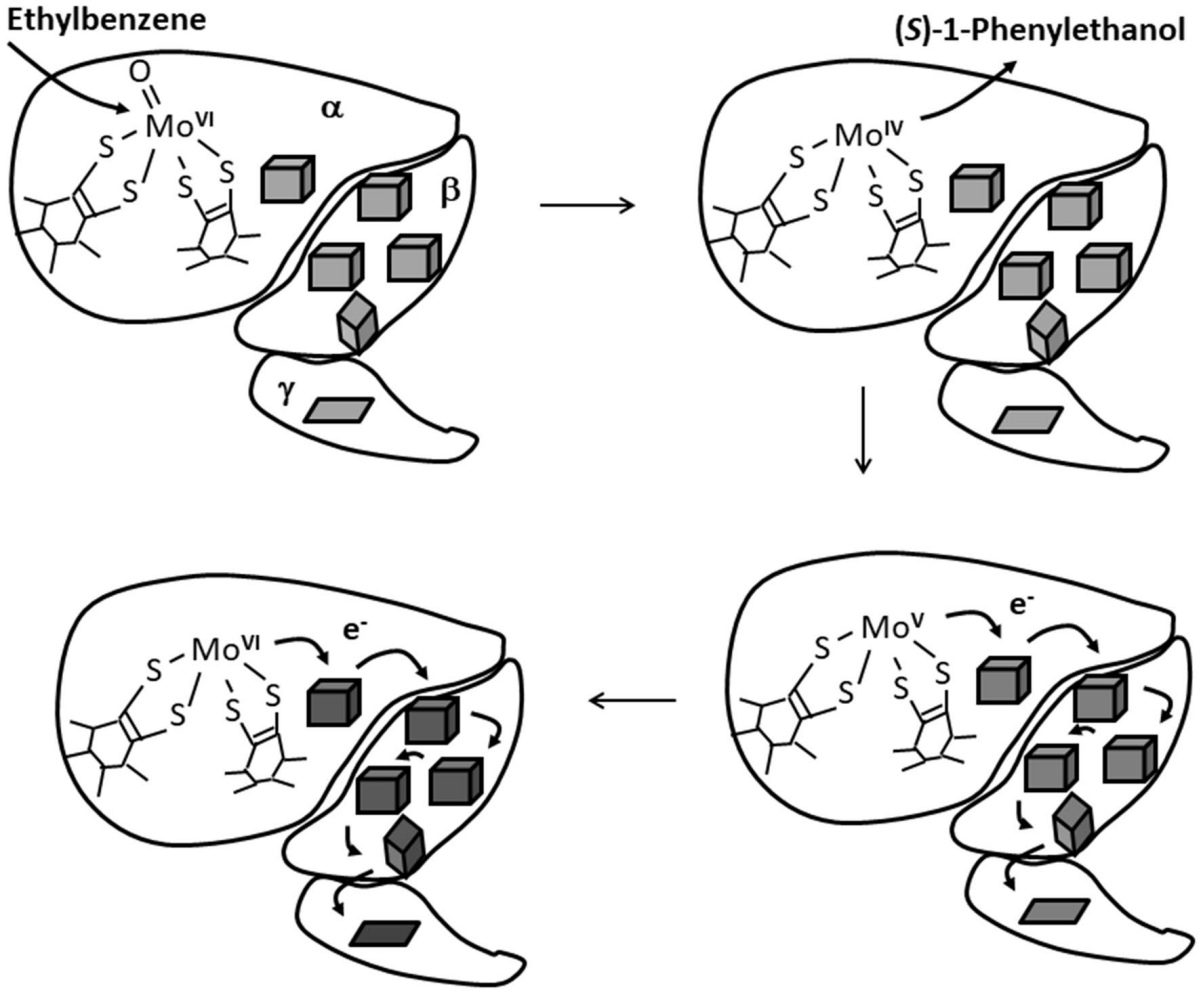
Reaction of ethylbenzene dehydrogenase. The three subunits are shown with the respective redox-cofactors contained and labelled as α, β and γ, respectively. The partial Mo-cofactor in the large α-subunit is shown in the oxidised (Mo^VI^) and reduced state (Mo^IV^), [4Fe–4S]-clusters are shown as large cubes, the [3Fe–4S]-cluster as smaller cube, and the heme *b* cofactor as a trapezoid. The electrons migrate through the cofactors one by one and are released to external acceptors from the heme *b* site in the γ-subunit

Ethylbenzene dehydrogenase (EbDH) is the initial enzyme of anaerobic ethylbenzene degradation in the denitrifying beta-proteobacterium *Aromatoleum* *aromaticum* [1–3]. It is a soluble molybdenum enzyme localised in the periplasmic space, consists of three subunits in an αβγ-complex and catalyses an oxygen-independent and stereospecific hydroxylation of ethylbenzene to (*S*)*-*1-phenylethanol as shown in Fig. 1 [4, 5]. Anaerobic hydroxylation of non-activated hydrocarbon groups is a highly unusual reaction in biochemistry and has also been described for other closely related periplasmic molybdenum enzymes acting on sterol side chains [6–10] or the methyl group of *p*-cymene [11]. Together with a number of further related periplasmic enzymes such as dimethylsulfide or steroid dehydrogenases, selenate, chlorate and perchlorate reductases, as well as membrane-bound nitrate reductases, these enzymes constitute a separate group (subfamily II) among the molybdenum enzymes of the “DMSO reductase family” [2]. A distinct feature of all members of subfamily II is a universally conserved aspartate, which serves as a ligand of the Mo atom in the active site [2, 12–19].

EbDH was crystallised under anaerobic conditions and its structure was solved at a resolution of 1.88 Å [18]. The architecture and cofactor content in the α and β subunits are highly similar to those of the corresponding subunits of the other two structurally characterised enzymes of the subfamily, nitrate reductase of *Escherichia* *coli* (NarGHI and NarGH) [16, 17] and perchlorate reductase of *Azospira* *suillum* (PcrAB) [20]. The structure of the γ subunit of EbDH is common to most other members of subfamily II, except for NarGHI and perchlorate reductase, displaying a single heme *b* cofactor in an unusual ligation by a lysine and a methionine [18]. In case of nitrate reductase, the γ subunit (NarI) is an integral membrane protein with two heme *b* cofactors [16], whereas perchlorate reductase contains a soluble γ subunit with a heme c-cofactor [21, 22], which is missing in the structurally characterised complex [20]*.*

**Fig. 2:**
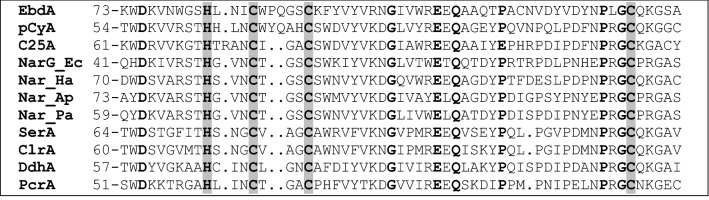
Sequence alignment of the N-terminal cysteine-clusters of some molybdenum enzymes of DMSO reductase subfamily II. *EbdA* ethylbenzene dehydrogenase, *pCyA* *p*-cymene hydroxylase, *C25A* cholesterol-C25 hydroxylase, *NarG* membrane-bound respiratory nitrate reductase, *Nar* periplasmatic nitrate reductase, *SerA* selenate reductase, *ClrA* chlorate reductase, *DdhA* dimethylsulfide dehydrogenase, *PcrA* perchlorate reductase. Abbreviations for organisms are: *Ap* *Aeropyrum* *pernix*, *Ec* *Escherichia* *coli*, *Ha* *Haloarcula* *marismortui*, *Pa* *Pyrobaculum* *aerophilum*. Conserved residues are in bold, the ligands of [Fe–S]-cluster FS0 are underlayed with grey

The α-subunit of EbDH harbours the catalytic center with the molybdenum cofactor, which is in a reduced (Mo^IV^) form in the known structure [18]. As suggested by theoretical modelling studies and experimental data on kinetic effects with different substrates or inhibitors, ethylbenzene appears to hydroxylated at the oxidised Mo-cofactor (Mo^VI^ form) by initial withdrawal of one electron and a proton, forming a radical transition state [23–25]. This is then followed by the withdrawal of the second electron to yield a carbocation-state intermediate, followed by a hydroxyl rebound reaction from the Mo center to yield the product (*S*)-1-phenylethanol [23, 24]. The Mo-cofactor is simultaneously reduced to the Mo^IV^ form, which needs to be re-oxidised by diverting single electrons from the molybdenum cofactor via the five iron–sulfur clusters (FS0–FS4) to the heme *b* cofactor (Fig. 1). The [4Fe–4S]-cluster FS0 is located in the α-subunit close to the active site and exhibits an unusual ligand architecture with three cysteines and one histidine [18], whereas the [4Fe–4S]-clusters FS1–FS3 and the [3Fe–4S]-cluster FS4 exhibit conventional cysteine ligands and are located in the β-subunit. Remarkably, nitrate reductase and perchlorate reductase show almost identical patterns of Fe–S cluster ligands in the α and β subunits as seen in EbDH [16–18, 20]. In particular, an unusual histidine ligand of cluster FS0 is apparently fully conserved in all enzymes belonging to subfamily II (Fig. 2).

The redox properties of the cofactors involved in electron transfer in enzymes of subfamily II have so far been reported for nitrate reductase [14, 26, 27], selenate reductase [28] and dimethylsulfide dehydrogenase [29, 30]. All of these enzymes have in common a relatively low midpoint potential of the second [Fe_4_S_4_] cluster of the β-subunit, whereas the potentials of the other redox cofactors vary between the enzymes [14, 28, 30]. In this paper, we present redox titration experiments on ethylbenzene dehydrogenase followed by EPR- and UV–Vis spectroscopy and compare the results to those of the other characterised molybdenum enzymes of subfamily II. These data contribute to the further elucidation of the mechanisms of electron flow from the catalytically active Mo-cofactor to the heme *b* implied in electron exit.

## Experimental procedures

### Growth of bacteria, purification and assays of ethylbenzene dehydrogenase

Growth of *Aromatoleum* *aromaticum* strain EbN1 and purification of ethylbenzene dehydrogenase were performed as described previously [18, 31, 32]. The catalytic activity of ethylbenzene dehydrogenase was measured as the decrease of absorption of ferricenium at 290 nm as previously described [4, 31, 32], except that the assay was performed at the optimum temperature of ethylbenzene dehydrogenase of 55 °C and that 200 µM ferricenium tetrafluoroborate (Aldrich) was used as electron acceptor instead of ferricenium hexafluorophosphate.

### Redox potentiometry and EPR spectroscopy

Redox titrations were carried out under argon atmosphere in a closed glass vessel [33] at 25 °C in 10 mM Tris–HCl, pH 8.0, 10% glycerol, 100 µM ferricenium tetrafluoroborate buffer in the presence of following redox mediators (20 µM concentration each): potassium hexacyanoferrate (III), dimethyl paraphenylene diamine, 1,1-dimethylferrocene, tetramethyl paraphenylene diamine, 2,6-dichlorophenol indophenol, 1,2 naphthoquinone, trimethyl-hydroquinone, vitamin K3 (menadione), 2-hydroxy-1,4-naphthoquinone, vitamin K1 (2-methyl-3-phytyl-1,4-naphthoquinone), anthraquinone-2-sulfonate, neutral red, benzyl viologen dichloride, methyl viologen hydrate, 2,4 dinitrophenol. The enzyme concentration used was 3.5 mg ml^−1^. Potentials were adjusted by adding small amounts of saturated sodium dithionite solution and measured with an Ag/AgCl KCl (3 M) and a Pt-indicator electrode, which were calibrated using a cytochrome c sample. All values were converted from potentials versus the reference electrode to those versus the standard hydrogen electrode by adding 208 mV and are given as such in the following text. Stable potentials were achieved a few minutes after dithionite additions, and frozen EPR samples were prepared within less than 5 s: the samples were directly transferred into calibrated argon gas-filled EPR tubes through a rubber septum, using an argon-flushed syringe, then the tubes were immediately frozen in a methylcyclohexane/isopentane mixture (1:5; v/v) chilled by liquid nitrogen and stored at – 80 °C until used. EPR spectra were recorded using a Bruker EMX 6/1 X-band spectrometer equipped with an orthogonal standard TE 102 microwave cavity and an ESR-900 helium evaporating cryostat (Oxford Instruments, Oxford, UK), which allows measurement at distinct temperatures (10–57 K). Spectra were recorded under non-saturating conditions at 12 K and 60 K.

### Electrochemically induced redox difference spectroscopy

UV–vis spectra between 200 and 600 nm were recorded in a UV–vis diode array photometer (J&M, Aalen, Germany) as a function of the applied potential. A homemade electrochemical thin-layer cell with an optical-transparent window composed of quartz glass (sample volume 3 µl, optical path length 5 µm) was used, which consisted of a three-electrode arrangement [34]. The sample is sealed between the glass plates under anoxic conditions and no oxygen can enter the cell [35]. A gold-grid disk was used as working electrode, a Pt spade electrode as auxiliary electrode, and a calomel electrode as reference electrode. Electrochemical analysis was performed at 6 °C with highly concentrated ethylbenzene dehydrogenase (87 mg ml^−1^) in buffer containing 10 mM Tris–acetate pH 8, 100 mM potassium chloride, 160 mM 100 µM ferricenium tetrafluoroborate and 10% glycerol. The following redox mediators were added (45 µM concentration each): tetramethyl paraphenylene diamine, 2,6-dichlorophenol indophenol, 1,2 naphthoquinone, tetramethyl hydroquinone, vitamin K3 (menadione), 2-hydroxy-1,4-naphthoquinone. Because of the short light path in the optical cell, redox-dependent absorption changes of the mediators are below the detection limit [35]. Redox potentials were adjusted gradually in steps of 25 mV, starting at a potential of + 137 mV versus the standard hydrogen electrode (NHE). The UV–vis spectrum of the protein at + 137 mV was used as reference for difference spectra. The potential was increased up to + 387 mV. Equilibration times between recording the UV–vis spectra were constant and added up to approximately 10 min. For control, the enzyme was re-reduced to + 137 mV and the UV–vis spectrum was compared to that recorded in the beginning. Baselines were corrected based on the absorbance at 600 nm and the spectra were normalized to the reference spectrum based on the absorbance at 280 nm. Difference spectra were obtained by subtracting the reference spectrum. To obtain the midpoint redox potential of the heme *b* cofactor, absorption value differences were plotted as a function of the applied potential. The amplitudes of the peaks at 424 nm, 528 nm and 559 nm were then fitted against the Nernst equation by the program package Origin Pro 9.0 (Origin Cooperation, Northampton, MA, USA) [36] assuming a one-electron transfer.

## Results

### Redox potentials of iron–sulfur clusters

**Fig. 3:**
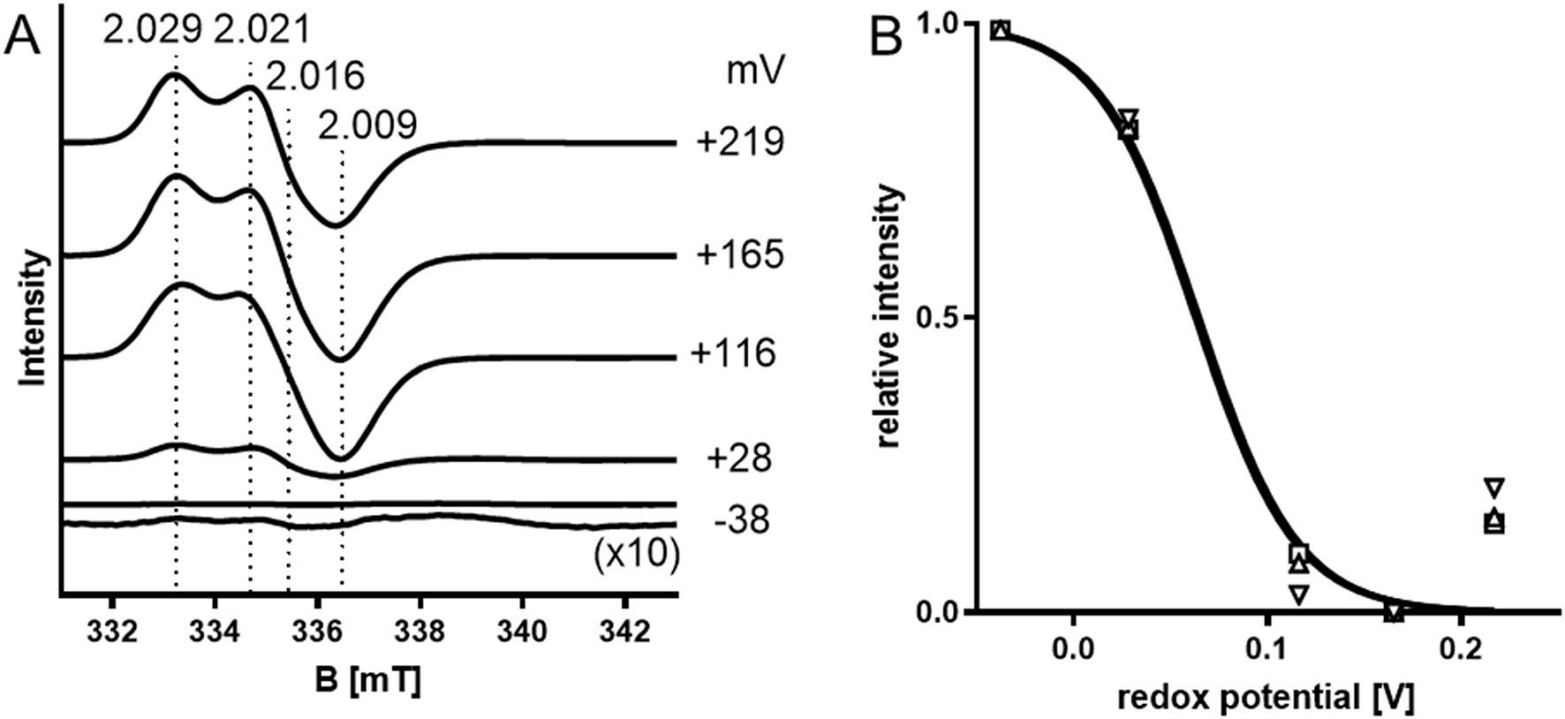
EPR spectra of EbDH at redox potentials ranging from + 219 mV to – 38 mV. Purified ethylbenzene dehydrogenase mixed with redox mediators (3.5 mg protein ml^−1^) was poised at the indicated redox potentials by adding small aliquots of a sodium dithionite stock solution. **A** EPR spectra recorded at 12 K under non-saturated conditions from samples taken after equilibrating at the respective potentials. Redox potentials are denoted versus the standard hydrogen (SHE) electrode. The EPR spectrum recorded for the sample at – 38 mV is additionally shown in tenfold enlargement to visualise the spectral features. The *g* values of important signals are indicated. **B** Curve fitting of the relative signal amplitudes at *g* = 2.0029, 2.021 and 2.009, exhibiting an average midpoint potential of + 70 ± 7 mV. Spectra were recorded under the following experimental conditions: microwave power, 10 mW; microwave frequency, 9.46589 GHz; modulation frequency, 100 kHz; modulation amplitude, 0.6 mT; 1 scan

EbDH was purified from ethylbenzene-grown cells of *A.* *aromaticum* in the presence of the electron acceptor ferricenium tetrafluoroborate as described previously [32]. Under these conditions, the enzyme stays in the oxidised form and is resistant to exposure to air, while reduced EbDH is readily inactivated by oxygen [4]. As the single [3Fe–4S]-cluster of EbDH was expected to be EPR-active in the oxidized form, while the [4Fe–4S]-clusters should only become EPR-active upon reduction, we initially compared the EPR spectra of purified EbDH and EbDH after reduction with dithionite. The obtained EPR signals at temperatures below 20 K were typical for a [3Fe–4S]-cluster containing protein with EbDH as isolated, but a number of very different signals were recorded for various dithionite-reduced samples, suggesting more complex changes in the EPR spectrum during reduction than the expected disappearance of a [3Fe–4S]^1+^ signal and appearance of [4Fe–4S]^1+^ signals (data not shown). Therefore, we performed redox titration on EbDH, poising the enzyme at different defined redox potentials and adjusting it to several redox states. This analysis allowed to follow the redox behaviour of most of the Fe–S clusters of EbDH and to determine approximate midpoint potentials for their redox changes. The oxidised form of EbDH as isolated showed a redox potential of + 219 mV versus NHE and exhibited an axial EPR spectrum (recorded at 12 K) consistent with a typical [3Fe–4S]^1+^ cluster with *g*-values at *g*_*x*_ = 2.029 and *g*_*y,z*_ = 2.016 (Fig. 3). The shape of the signal is similar to those of the [3Fe–4S]^1+^ iron–sulfur clusters (FS4) of DMS dehydrogenase DmsABC [30], selenate reductase SerABC [29] and nitrate reductase NarGHI [26], although the g values do not match exactly. Stepwise reduction of the enzyme with sodium dithionite to a redox potential of – 38 mV (versus NHE) led to the expected disappearance of the [3Fe–4S]^1+^ signal and the appearance of new signals correlated to [4Fe–4S]^1+^ clusters (Fig. 3). The [3Fe–4S]^1+^ cluster was apparently fully oxidised at redox potentials higher than + 165 mV and disappeared at redox potentials lower than − 38 mV, indicating its full reduction (Fig. 3). The signal amplitudes at g values of 2.029, 2.021 and 2.009 were plotted as functions of the applied potentials and fitted to the Nernst equation, assuming a one-electron-transfer (Fig. 3B), yielding an averaged midpoint redox potential value of + 70 ± 7 mV for the [3Fe–4S] cluster of ethylbenzene dehydrogenase.

**Fig. 4:**
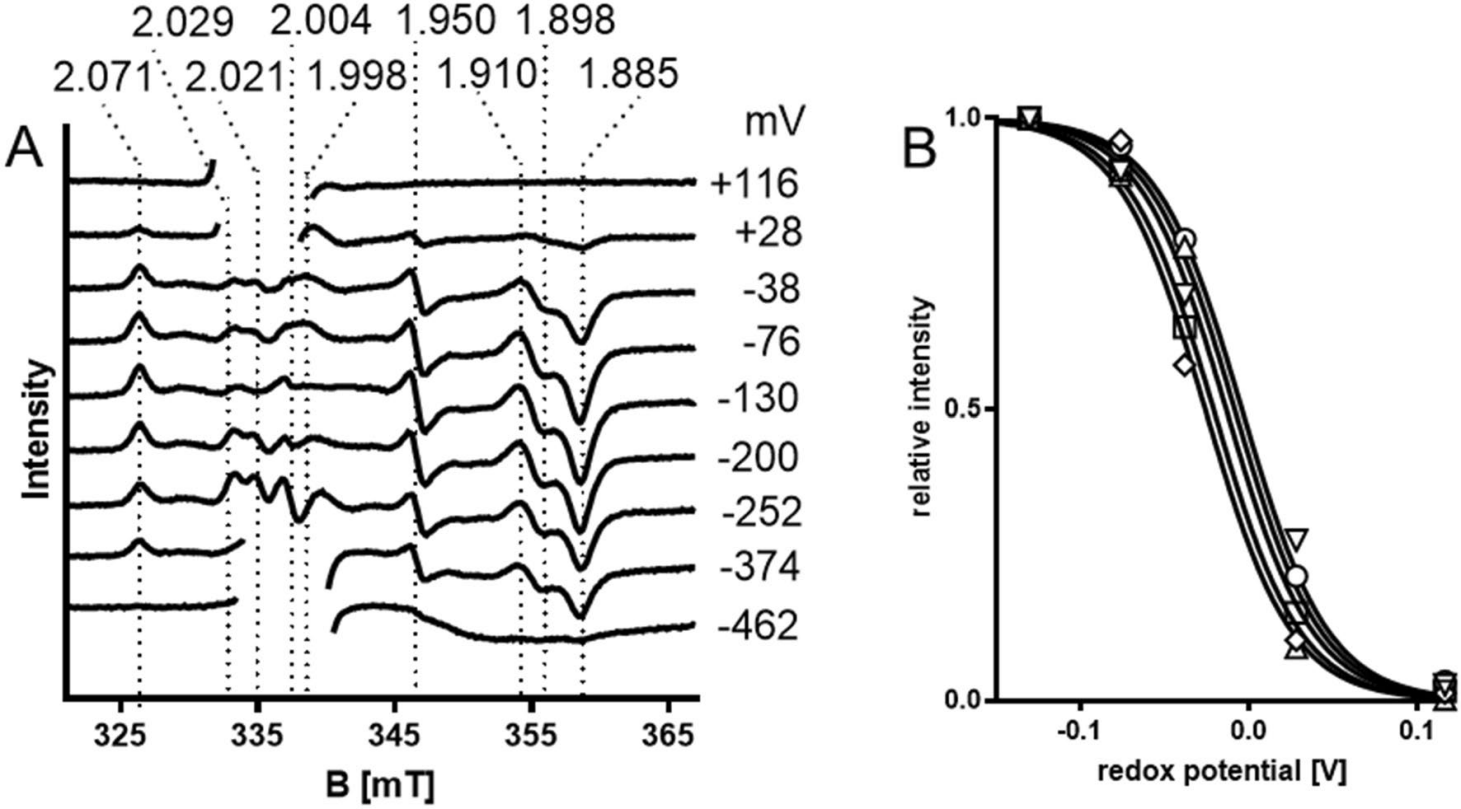
**A** EPR spectra of EbDH at redox potentials ranging from + 116 mV to – 462 mV. EPR conditions were identical as for Fig. 3. For better identification of the signals, resonances of the [3Fe–4S] cluster were cut off between *g* = 2.040 and 1.998 (equivalent to 332–339 mT) in the spectra between + 116 mV to + 28 mV, as well as the resonances corresponding to the mediator-derived radical signal around *g* = 2.0 in the spectra at – 374 mV and – 462 mV. The *g*-values of prominent signals are indicated. **B** Curve fitting of the relative signal amplitudes at *g* = 2.071, 1.950, 1.910, 1.898 and 1.885, exhibiting an average midpoint potential of − 18 ± 9 mV

At redox potentials between – 38 and –372 mV, the EPR spectra of ethylbenzene dehydrogenase showed typical resonances of [4Fe–4S]^1+^ clusters at *g* values of 2.071, 1.950, 1.910, 1.898 and 1.885 (Fig. 4). These resonances were expected to correlate to the [4Fe–4S]^1+^ clusters FS1 and FS3 of ethylbenzene dehydrogenase and their midpoint redox potential values ranged between—5 and – 26 mV after fitting the respective signal amplitudes with the Nernst equation (Fig. 4B). Spectral features between *g* = 2.029 and 1.998 could not be evaluated because of the rise of a strong radical signal from the added redox mediators with low redox potentials (Fig. 4). Because of the close proximity of the recorded midpoint potentials, we assume a very similar redox behaviour of FS1 and FS3, but we are unable to attribute the signals to either of these two Fe–S clusters. Averaging the midpoint potentials determined for the non-disturbed resonances (e.g. those close to *g* = 2, where the radical signals of the viologens start to rise) provided values of – 18 ± 9 mV for the midpoint redox potentials of FS1 and/or FS3. Comparing the obtained spectra to those of DMS dehydrogenase, selenate reductase and nitrate reductase (NarGHI) reveals resonances at similar *g* values, but the spectra are too divergent to correlate spectral features with the corresponding Fe–S clusters [14, 27]. A clear annotation of the individual iron–sulfur clusters with the observed EPR signals will require the production of mutant variants with inactivated Fe–S clusters in recombinant ethylbenzene dehydrogenase, which still needs to be established experimentally.

Upon reduction of EbDH beyond − 400 mV, the shape of the EPR spectrum changed significantly. All resonance peaks started to diminish below − 300 mV, and at − 462 mV an extremely broadened and unstructured spectrum was recorded that did not contain any of the previously visible peaks (Fig. 4). This behaviour explains the initially observed problems in recoding the EPR spectrum of reduced EbDH, because any intermediate state might have been obtained without control of the redox potential, depending on the respective enzyme and dithionite concentrations used. A similar behaviour was reported for the EPR spectra of DmsABC, SerABC and NarGHI, which lost a number of well-defined resonances of reduced [4Fe–4S] clusters at redox potentials < − 400 mV, but still exhibited a distinctive shape with strong resonances at very low potentials[14, 27–29]. In all cases, the partial or total loss of apparent EPR peaks can be explained by assuming that cluster FS2 is only reduced at very low redox potentials and then instigates magnetic coupling with the reduced neighboring clusters FS1 and FS3, resulting in broadening of the signals. The overall shape of the spectrum recorded for EbDH and the presence of broad resonances around *g* ≈ 1.9 (Fig. 4) are typical for spectra of [4Fe–4S]^1+^ clusters magnetically coupled to other iron–sulfur clusters [27]. The very short distances between FS1 and FS2 (6.6 Å) and between FS2 and FS3 (5.4 Å) known from the crystal structure of EbDH [18] corroborate this interpretation and may be responsible for the apparently very strong magnetic coupling effect in EbDH. Therefore, it seems that clusters FS1 and FS3 act as independent EPR-active centers only as long as cluster FS2 remains in the oxidised (diamagnetic) state. Consequently, the observed broadening of the spectrum of EbDH indicates that the redox midpoint potential of cluster FS2 is between − 374 and − 462 mV, which is in the same range as the redox potential of the other studied proteins of the enzyme subfamily (− 337, − 336, and − 420 mV for the FS2 clusters of DmsABC, SerABC and NarGHI, respectively) [14, 27–29].

The unusually ligated iron–sulfur cluster FS0 of nitrate reductase A was characterised as an iron-sulfur cluster with an *S* = 3/2 high-spin ground state, resulting in EPR resonances at *g* = 5023 and *g* = 5556 [14]. However, even after careful inspection of our EPR spectra, we never detected any unusual signals for EbDH that could be correlated to FS0 or any other potential redox cofactor (data not shown). The same has been reported for DmsABC and SerABC [28, 29], suggesting that EPR resonances of FS0 are only visible under special experimental conditions.

### Redox potentials of the molybdenum cofactor

**Fig. 5:**
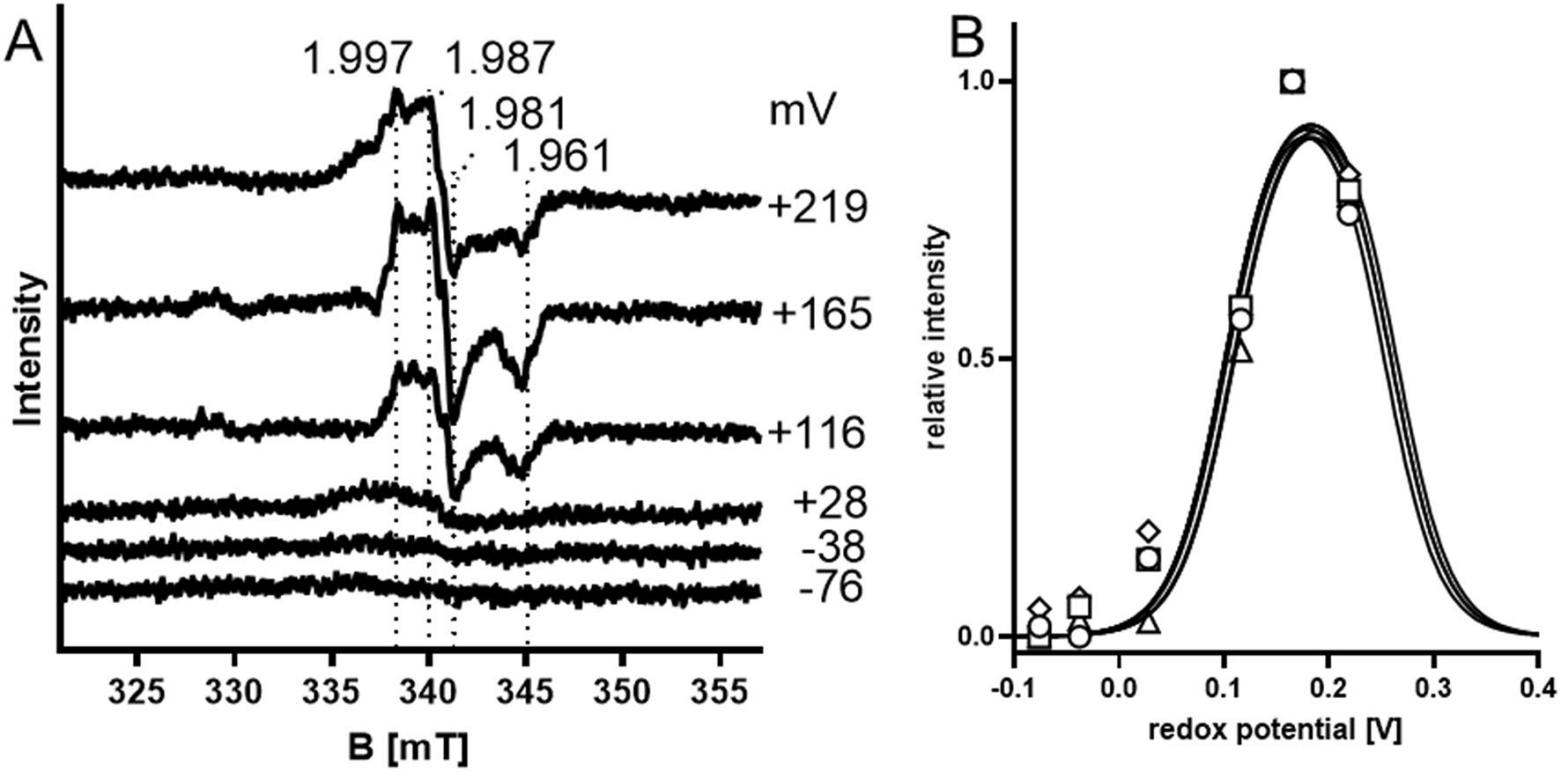
**A** Mo^V^ EPR signals of ethylbenzene dehydrogenase at 60 K and redox potentials from + 219 mV to – 76 mV. The conditions were the same as for Fig. 3 except that the temperature was 60 K, the microwave power was 5 mW and the modulation frequency was 0.4 mT. The *g*-values of important signals are indicated. **B** Curve fitting of the relative signal amplitudes at *g* = 1.997, 1.987, 1.981 and 1.961. The calculated average midpoint potential of Mo^IV^/Mo^V^ was at + 99 ± 10 mV, whereas that of Mo^V^/Mo^VI^ was extrapolated at 259 ± 20 mV by averaging the values for all four major resonances

EPR signals of paramagnetic Mo^V^ species in EbDH were recorded at temperatures of 60 K to discriminate them against those of the quickly relaxing spin systems of the Fe–S clusters, which are only detectable at temperatures below 40 K. Under these conditions, a rhombic signal was visible at *g* values of 1.997, 1.987, 1.981 and 1.961 in samples taken at redox potentials from + 219 mV to − 76 mV (Fig. 5). The spectral features are similar with the signals attributed to Mo(V) in DmsABC, SerABC and NarGHI [26, 28, 29]*.* The relative amplitudes of the observed peaks were plotted against the applied redox potentials, showing bell-shaped graphs with maxima around + 183 mV (Fig. 5B). Fitting these data with the Nernst equation (assuming two consecutive one-electron redox processes) yielded average values of the midpoint redox potentials for Mo^VI//V^ of + 259 ± 20 mV and Mo^V/IV^ of + 99 ± 15 mV (Fig. 5B). Unfortunately, the highest available redox potential in our study was at + 219 mV (corresponding to ferricyanide as oxidant), where we still recorded a strong Mo(V) signal, leading to some uncertainty of the extrapolated Mo^VI//V^ midpoint potential. However, since all four of the main Mo^V^ resonances showed almost identical behaviour, we are confident that the determined value is close to the actual Mo^VI//V^ midpoint potential.

### Redox potential of the heme ***b*** cofactor

**Fig. 6:**
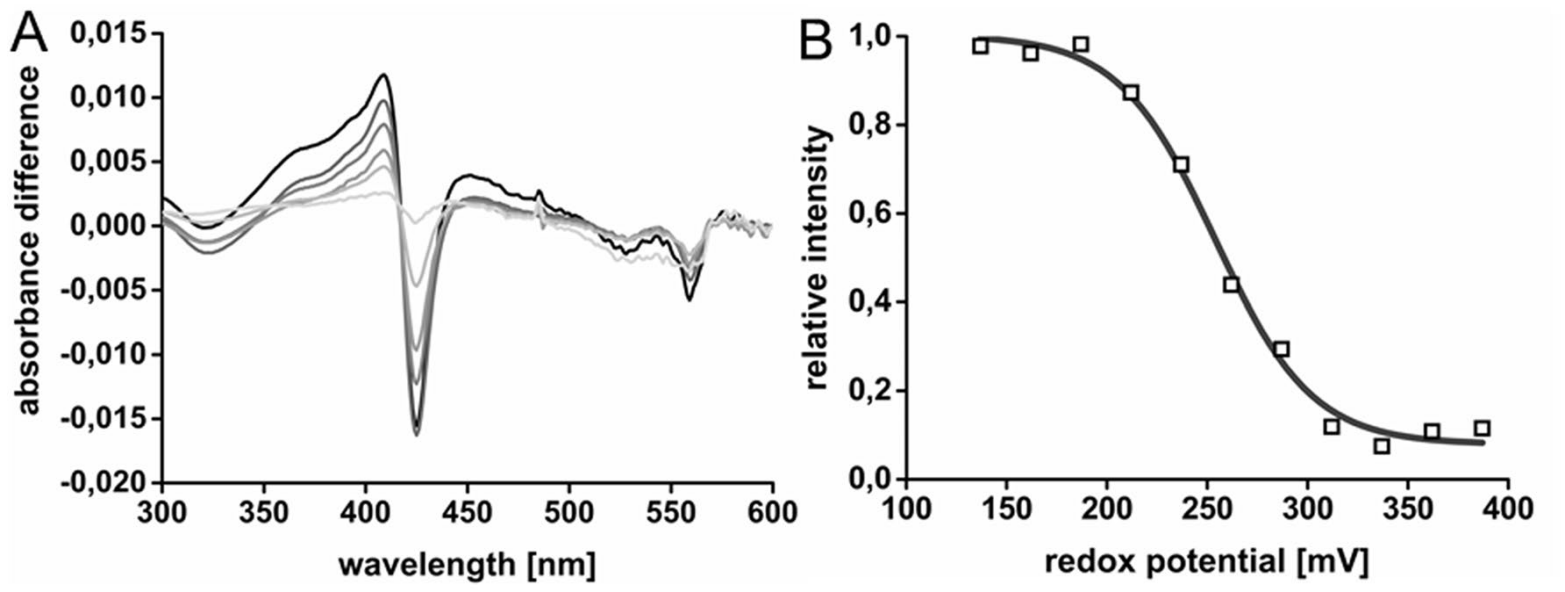
**A** Difference UV–Vis spectra of EbDH at different redox potentials. The spectrum recorded at + 137 mV showed a completely reduced heme *b* cofactor and was used as reference for difference spectra with the enzyme at higher redox potentials up to + 287 mV (light to dark grey). **B** Fitting of the difference peak at 424 nm to the Nernst equation, assuming a one-electron transfer

The midpoint potential of the heme *b* cofactor of ethylbenzene dehydrogenase was defined via electrochemically induced redox difference spectroscopy [37] (see experimental procedures). The experiment was based on recording the blue-shift of the Soret band from 424 to 414 nm correlated with oxidising the heme *b* cofactor. Comparison of the UV–vis spectra of ethylbenzene dehydrogenase showed that the heme *b* cofactor was completely reduced at + 137 mV and became fully oxidised at a redox potential of + 387 mV, as judged by the movement of the Soret band from 424 to 414 nm and the disappearance of the peaks at 520 and 559 nm (Fig. 6). Oxidation and reduction of the heme *b* was fully reversible (data not shown). The midpoint redox potential of the heme *b* cofactor was determined from plots of the signal amplitudes of the difference spectra against the applied potentials and curve fitting to the Nernst equation (Fig. 6B). Assuming a one-electron-transfer, an average midpoint potential of the heme *b* cofactor of 256 ± 0.7 mV was obtained. As for all other midpoint potential determinations reported here, only one-electron-based equations yielded good fits to the experimental data, whereas curve fits assuming two-electron transfer were not compatible with the recorded data.

## Discussion

We investigate here the redox properties of the metal cofactors of EbDH, which mediate electron transfer through the enzyme from the molybdenum cofactor in the active site to the heme *b* as putative electron exit site. Their properties are discussed in the order of the presumed electron flow.

### Molybdenum cofactor

Ethylbenzene dehydrogenase contains a molybdenum-bis-MGD cofactor located in the α-subunit and forms an additional bond to the Mo atom via aspartate residue 223, which is conserved in all enzymes of subfamily II of the DMSO reductase family [18]. This cofactor is part of the active site and constitutes the site of substrate hydroxylation and uptake of two electrons, which are subsequently channelled through the enzyme to a heme *b* cofactor in the γ-subunit. The midpoint potentials of the Mo^VI^/Mo^V^ and Mo^V^/Mo^IV^ transitions of EbDH of + 259 mV and + 99 mV, respectively, are very similar to those of reported for nitrate reductase NarGHI (+ 200 mV for Mo^VI^/Mo^V^ and + 100 mV for Mo^V^/Mo^IV^), although the reactions catalysed by these enzymes occur in opposite directions [14]. The only related enzyme catalysing an oxidative reaction with characterised midpoint potentials, dimethylsulfide dehydrogenase, shows lower potentials for the Mo-cofactor [29], presumably reflecting the different prerequisites involved in dimethylsufide vs. hydrocarbon oxidation. While most other molybdenum enzymes catalyse hydroxylation or reduction of their substrates in a two-electron redox process [38, 39], ethylbenzene hydroxylation is proposed to operate via an initial one-electron transfer from the substrate to the Mo-cofactor, which is predicted to be the rate-limiting step [23–25]. Therefore, a high redox potential for the Mo^VI^/Mo^V^ transition providing a large driving force for the first step seems consistent with the mechanistic proposal. Transfer of the second electron is predicted to be coupled with hydroxylation of the intermediate [24] and should therefore require a significantly lower redox potential for the Mo^V^/Mo^IV^ transition.

### Iron–sulfur clusters

The crystal structure of ethylbenzene dehydrogenase showed that five iron–sulfur clusters (FS0–FS4) are present in ethylbenzene dehydrogenase [18], which show an almost identical ligation and geometry as in NarGHI [16, 17]. One of them (FS0) is located in the alpha subunit and four (FS1-4) are located in the beta subunit. The locations and distances of these clusters within the enzyme structure show that FS0 links the electron transfer from the Mo-cofactor to the Fe–S clusters of the β-subunit. All distances between the chain of redox cofactors in EbDH are shorter than 9 Å [18], which should allow a highly efficient flow of electrons through the enzyme via tunnelling [40]. Moreover, the electrons are clearly moving through the Fe–S clusters in numerical order from FS0 to FS4 and finally end up at the heme *b* cofactor of the γ-subunit (Fig. 1).

The unusually ligated FS0 cluster was reported in NarGHI to be associated with an unusual high-spin ground state [14], but was not yet visualised spectroscopically in EbDH or any other related enzyme. As deduced from the similarities of the structures and conserved sequences [14, 18], the same type of FS0 ligation by three Cys and a His seems to be conserved in all enzymes of DMSO reductase subfamily II, including the recently described cholesterol C25 hydroxylase and *p*-cymene methylhydroxylase (Fig. 2) [2]. However, we cannot conclude that the high-spin ground state is conserved throughout all enzymes of the subfamily. If FS0 of EbDH shows a *S* = ½ state in its reduced form, correlated EPR signals might be contained in the complex spectrum recorded between redox potentials of − 38 to − 374 mV (Fig. 4).

The EPR signals in the − 38 to − 374 mV redox potential range appeared after the EPR signal of the [3Fe–4S]^+1^ cluster just vanished and represent more than one reduced [4Fe–4S] ^**1+**^-cluster (Fig. 4). All observed spectral features behave very similarly in respect to their midpoint potentials (between − 5 and − 26 mV) and magnetic saturation behaviour, precluding a definitive correlation of EPR peaks to individual Fe–S clusters. We expect that the observed spectral features represent mostly the reduced [4Fe–4S] ^**1+**^-forms of the regularly ligated clusters FS1 and FS3 and assume a common midpoint potential of both clusters at around − 18 mV. A clear distinction of the properties of the [4Fe–4S]-clusters will only be possible by analysing mutated EbDH variants, which is one of the aims of our ongoing efforts to establish recombinant expression of EbDH. The values attributed to FS1 and FS3 differ significantly from those of the corresponding clusters in related molybdenum enzymes (Table 1).

**Table 1:** Midpoint redox potentials of the redox cofactors of molybdenum enzymes

Protein	Mo^VI/V^	Mo^V/IV^	FS0	FS1	FS2	FS3	FS4	heme *b*1/ heme *b*2	References
EbDH	+ 259	+ 99	nd	– 18	~ – 400	– 18	+ 70	+ 256	This paper
Dms-DH	+ 123	+ 56	nd	+ 175	– 337	+ 66	+ 292	+ 324	[29, 30]
Ser	nd	nd	nd	+ 183	– 336	– 51	+ 118	nd	[28]
NarGHI	+ 200	+ 100	-55	+ 130	– 420	– 55	+ 180	+ 120/ + 20	[14, 26]
NarGH	nd	nd	nd	+ 60	– 400	– 200	+ 80	nd	[27]

EbDH is compared to the related periplasmic enzymes dimethylsulfide dehydrogenase (Dms-DH) and selenite reductase (Ser), the membrane-bound nitrate reductase (NarGHI) and a soluble nitrate reductase variant lacking the membrane anchor subunit (NarGH). Potentials are given in mV vs. the NHE at pH 7. All enzymes are members of DMSO reductase subfamily II

*nd* Not detected

Cluster FS2 of ethylbenzene dehydrogenase is apparently only reduced to an EPR-active form at an extremely low redox potential of around − 400 mV, as reported for all other related enzymes [20, 26–30, 41]. The distances from FS2 to FS1 and to FS3 are the shortest between any redox cofactors in EbDH [18], which is consistent with the strong magnetic coupling of the signals of the three reduced Fe–S clusters. This effect leads to the observed extreme broadening and apparent disappearance of the EPR signals as soon as FS2 becomes reduced (Fig. 4). Similar broadened EPR spectra from magnetic coupling of clusters FS1–FS3 were reported for NarGHI at similar redox potentials (< − 400 mV), and for dimethylsulfide dehydrogenase and selenate reductase at slightly higher redox potentials (< − 350 mV) [20, 27–30, 41]. The low redox potential of the FS2 cluster was interpreted as a potential barrier for preventing any uncontrolled passage of electrons through the enzyme [14, 26].

The [3Fe–4S] ^1+^ cluster of the beta subunit (FS4) was assessed most clearly in its properties, since it corresponds to the only detectable EPR signal in the oxidized state of the enzyme and showed a relatively high midpoint redox potential of + 70 mV. The FS4 clusters of the related enzymes show similar resonances, although the respective midpoint potentials are quite different and mostly more positive in the other characterised enzymes [14, 20, 26, 28–30, 41].

### Heme ***b*** cofactor

The determined midpoint potential of the heme *b* cofactor of ethylbenzene dehydrogenase is very high at + 256 mV. A higher value has only been reported for dimethylsulfide dehydrogenase (+ 324 mV) among the related enzymes, whereas the values of the heme *b* cofactors of NarGHI are much lower and fit to their function of receiving electrons from menaquinol or ubiquinol (Table 1). The high redox potential of the heme *b* cofactor of EbDH may be explained by the non-polar heme *b*inding pocket of the gamma subunit and the unusual ligation of the heme iron by methionine and lysine as axial ligands [18], which is conserved in dimethylsulfide dehydrogenase. Moreover, the high redox potential fits very well to previous biochemical data of ethylbenzene dehydrogenase, showing that ethylbenzene oxidation is only possible with artificial electron acceptors with high redox potentials (e.g. ferricenium, Fe(CN)_6_^3−^) [4, 42] or with cytochrome *c* [43], and to a direct electrochemical analysis of EbDH [44]. Furthermore, the heme *b* cofactor is located close to the surface of the protein and is supposed to act as electron donor to an external electron acceptor of similarly high potential [18]. Because ethylbenzene dehydrogenase is a periplasmic enzyme, a probable natural electron acceptor is cytochrome *c* of the respiratory chain, which has a midpoint potential of about + 350 mV.

Comparing EbDH to the other enzymes of DMSO reductase subfamily II with known redox potentials, it is evident that EbDH is the only enzyme with redox potentials higher than 250 mV at either end of the electron transfer chain, while the difference to NarGHI and Dms-DH is still not exceedingly high (Table 1). We propose that the high Mo^VI/V^ potential may be required to overcome the activation barrier associated with converting ethylbenzene to an initial radical intermediate. The redox potential difference between ethylbenzene/1-phenylethanol (30 mV) and Mo^VI/V^ (259 mV) would significantly contribute to achieve C–H activation of the substrate (calculated activation energy from QM–MM modelling of the EbDH reaction: 67.2 kJ/mol) [24]. The electrons have to migrate subsequently through the enzyme towards the heme *b*, which exhibits almost exactly the same midpoint potential (Table 1). This is achieved through a line of tightly coupled redox cofactors passing through a trough of very low redox potential within the enzyme, represented by steadily decreased midpoint potentials up to FS2, then again steadily increasing midpoint potentials towards the heme *b*. This enzymic property provides a thermodynamic barrier in the electron transfer path of EbDH and explains the original observation that reaction with ethylbenzene as reductant only led to full reduction of the heme *b* cofactor, while the Fe–S clusters needed further addition of dithionite to become fully reduced [4]. In accordance with earlier speculations, we propose that this feature allows more control over the reaction, because the electrons will only proceed beyond FS2 when “pushed” by further electrons coming from the active site and “pulled” by the release of electrons from the heme *b*. Therefore, the reaction will stop immediately when either the substrate or the electron acceptor is used up. Moreover, this thermodynamic barrier will prevent the backwards flow of electrons from the heme *b* to the active center.

## Data Availability

Not applicable.

## Abbreviations

EbDH: Ethylbenzene dehydrogenase
MGD: Molybdopterin guanine dinucleotide
DMSO: Dimethylsulfoxide
Nar: Nitrate reductase
EPR: Electron paramagnetic resonance
UV–Vis: Ultraviolet–visible light
NHE: Standard hydrogen electrode
QM-MM: Quantum mechanics/molecular modelling
Dms-DH: Dimethylsulfide dehydrogenase
Ser: Selenite reductase
Pcr: Perchlorate reductase
